# Recent advances in paclitaxel biosynthesis and regulation

**DOI:** 10.1093/jxb/erae240

**Published:** 2024-05-23

**Authors:** Toby Coombe-Tennant, Xiaoping Zhu, Shihua Wu, Gary J Loake

**Affiliations:** Institute of Molecular Plant Sciences, School of Biological Sciences, University of Edinburgh, The King’s Buildings, Edinburgh EH9 3BF, UK; Research Center of Siyuan Natural Pharmacy and Biotoxicology, College of Life Sciences, Zhejiang University, Hangzhou, Zhejiang Province 310058, China; Joint Research Centre for Engineering Biology, Zhejiang University–University of Edinburgh Institute, Zhejiang University, Haining 314400, China; Research Center of Siyuan Natural Pharmacy and Biotoxicology, College of Life Sciences, Zhejiang University, Hangzhou, Zhejiang Province 310058, China; Joint Research Centre for Engineering Biology, Zhejiang University–University of Edinburgh Institute, Zhejiang University, Haining 314400, China; School of Ecology and Environment, Tibet University, Lhasa 850000, China; Institute of Molecular Plant Sciences, School of Biological Sciences, University of Edinburgh, The King’s Buildings, Edinburgh EH9 3BF, UK; Joint Research Centre for Engineering Biology, Zhejiang University–University of Edinburgh Institute, Zhejiang University, Haining 314400, China; Green Bioactives Ltd, Douglas House, Pentland Science Park, Bush Loan Rd, Penicuik EH26 0PL, UK; University College Dublin, Ireland

**Keywords:** Cell culture, epigenetics, paclitaxel, plant specialized metabolites, regulation, taxol, *Taxus*

## Abstract

Paclitaxel (PTX) is a high value plant natural product derived from *Taxus* (yew) species. This plant specialized metabolite (PSM) and its derivatives constitute a cornerstone for the treatment of an increasing variety of cancers. New applications for PTX also continue to emerge, further promoting demand for this WHO-designated essential medicine. Here we review recent advances in our understanding of PTX biosynthesis and its cognate regulation, which have been enabled by the development of transcriptomic approaches and the recent sequencing and annotation of three *Taxus* genomes. Collectively, this has resulted in the elucidation of two functional gene sets for PTX biosynthesis, unlocking new potential for the use of heterologous hosts to produce PTX. Knowledge of the PTX pathway also provides a valuable resource for understanding the regulation of this key PSM. Epigenetic regulation of PSM in plant cell culture is a major concern for PTX production, given the loss of PSM production in long-term cell cultures. Recent developments aim to design tools for manipulating epigenetic regulation, potentially providing a means to reverse the silencing of PSM caused by DNA methylation. Exciting times clearly lie ahead for our understanding of this key PSM and improving its production potential.

## Teaser

Paclitaxel is a high value plant natural product with extensive medical uses. This expert view covers the elucidation of paclitaxel biosynthesis, its regulation and production in plant cell cultures.

## Introduction

Paclitaxel (PTX) is a high value plant specialized metabolite (PSM) of *Taxus* species produced as part of a jasmonate-dependent defence response against fungal pathogens. PTX is a member of the diterpenoid family (Gallego-Jara *et al*., 2020), whose structure was first elucidated in 1971 (Wani *et al*., 1971). In 2021, the global PTX market was valued at US$4.51 billion and is predicted to surpass US$11.16 billion in 2030 (Perez-Matas *et al*., 2022). This value stems from the widespread use of PTX in chemotherapy, due to its unique ability to both stabilize and promote the polymerization of microtubules (Schiff *et al*., 1979; Manfredi *et al*., 1982). PTX and related derivatives have been approved for treating multiple diseases such as breast, ovarian, pancreatic, non-small cell cancers, and Kaposi’s sarcoma (Yang *et al*., 2020). Furthermore, the potential applications of PTX have also been explored in the context of skin diseases (Bharadwaj *et al*., 2016), Alzheimer’s disease (Cross *et al.*, 2021), and cervical cancer (McCormack *et al.*, 2023). The widespread success of PTX, coupled with its expanding applications, has resulted in a high demand and associated high cost for this pharmaceutical, which makes accessibility to this drug a significant issue.

PTX was originally produced exclusively by natural harvesting, although this practice is non-sustainable (Malik *et al.*, 2011). PTX biosynthesis is not just limited to the genus *Taxus* and has been found in other tree species and endophytic fungi (Stierle *et al*., 1993; Hoffman *et al*., 1998), although yields from these are not yet viable for industrial production (Liu *et al*., 2016). Full PTX synthesis has been achieved by several routes (Holton *et al*., 1994; Nicolaou *et al*., 1994; Danishefsky *et al*., 1996). However, this is unsuitable for meeting the market demand, due to the required pathway being complex and expensive owing to the reagents and conditions necessary to achieve correct stereochemistry, resulting in overall yields being low (S. Zhang *et al.*, 2023). Production is possible through semi-synthesis from 10-deacetyl-bacatin III (10-DAB), a more abundant intermediate than PTX in *Taxus* species (Baloglu and Kingston, 1999).

An alternative viable approach for commercial PTX production is plant cell cultures (PCCs), first discovered as a means of PTX production in 1989 (Christen *et al*., 1991). PCCs are advantageous for use as biofactories due to being scalable, having cytochrome P450s and a supply of precursors (Wu *et al*., 2021), and their post-translational modification capabilities, being able to perform 11 of the 13 most common eukaryotic post-translational modifications (Coates *et al.*, 2022). PCCs offer sustainable PTX production that can alleviate pressures on biodiversity caused by natural harvest (Roberts, 2007), whilst also generating more consistent yields. One method for PSM yield improvement in PCCs is through the addition of ‘elicitors’ that function as plant immune activators, such as methyl jasmonate (MeJA) for PTX production (Mirjalili and Linden, 1996). These advantages have made PCCs commercially viable for a vast range of plant natural products and recombinant therapeutics (Ochoa-Villarreal *et al.*, 2016; Karki *et al*., 2021; Bapat *et al*., 2023).

PTX production in heterologous hosts is being explored as an alternative, but this was severely limited by the absence of a complete PTX pathway (Perez-Matas *et al*., 2023a). However, the publishing of three *Taxus* genomes in 2021 (Cheng *et al*., 2021; Song *et al*., 2021; Xiong *et al*., 2021) has led to the elucidation of genes encoding several missing steps in PTX biosynthesis. Subsequently, two breakthroughs have occurred through the transient expression of candidate genes in *Nicotiana benthamiana*, enabling the identification of a minimal gene set for PTX biosynthesis (Y. Zhang *et al.*, 2023) and an alternative gene set for baccatin III biosynthesis (Jiang *et al*., 2024).

With potential pathways for PTX production being established, our understanding of PTX biosynthesis and the underpinning molecular mechanisms that regulate this pathway can be deepened. PTX regulation research has long focused on the identification of elicitors, the first being derived from fungi (Ciddi *et al*., 1995). Subsequent studies examined the importance of transcription factors (TFs), such as TcWRKY1 (S. Li *et al.*, 2013). Nonetheless, current research into PTX biosynthesis regulation is centred on epigenetic modifications, which are shown to be a main contributing factor to decreased PSM production in older PCCs (Sanchez-Muñoz *et al.*, 2019b). This is problematic for maintaining high-yielding cell culture lines, which gradually lose PTX biosynthesis capacity when maintained long-term in *in vitro* cultures (Fu *et al*., 2012). Here we cover recent developments in PTX biosynthesis gene set identification and its regulatory mechanisms providing an updated resource for improving PTX production (Box 1).

Box 1. **Recent developments in PTX biosynthesis and regulation**

- Published genomes for *Taxus chinensis* (Xiong *et al*., 2021), *Taxus wallichiana* (Cheng *et al*., 2021) and *Taxus yunnanensis* (Song *et al*., 2021).
- Provides chromosomal-scale reference-grade genomes for each species enabling integrated approaches and detailed insights into the identification of the genes involved in PTX biosynthesis and its possible regulators and their mechanism of function.
- Investigated the impact of long-term cell culture on DNA methylation of three promoters of PTX biosynthesis genes in the loss of PTX production caused by long-term cell culture (Escrich *et al*., 2022).
- Highlighted the key role of DNA methylation in the loss of PTX production, further increasing our understanding of the regulation of the pathway and providing insight into potential new targets for decreasing this loss of function, which is associated with maintaining PCCs in *in vitro* conditions.
- Created a cell line using CRISPR-guided methylation to knock down a competitive pathway of PTX biosynthesis in *Taxus* cell cultures to increase PTX biosynthesis (Newton *et al.*, 2023).
- First application of CRISPR to *Taxus* PCCs, to achieve a 25-fold increase in PTX accumulation through the knockdown of PAL and use of chemical inhibitors to inhibit the competitive pathway, phenylpropanoid biosynthesis.
- Identification of missing enzymes enabling PTX and baccatin III biosynthesis (Zhang *et al*., 2023b; Jiang *et al*., 2024).
- Identification of two different routes for PTX biosynthesis providing valuable insight into the branching nature of PTX biosynthesis and providing new targets for investigating the controlling mechanisms of PTX biosynthesis. This also unlocks the potential of PTX production in heterologous hosts, as a full pathway can now be assembled.

## Genome-enabled enzyme identification

The absence of a *Taxus* chromosome-scale reference genome had limited the capability to perform genomic studies, thus limiting the understanding of the biosynthesis and regulatory mechanisms for PTX (Kui *et al*., 2022). This sentiment was emphasized with the publication of the *Taxus chinensis* genome with a comprehensive transcriptome covering eight tissues, two cell lines, and one cell line elicited with MeJA (Xiong *et al*., 2021). This greatly improved the resources available for determining the missing genes by providing gene locational detail. Ultimately, this led to the identification of a gene cluster for taxadiene biosynthesis, as well as a grouping of PTX biosynthesis genes on chromosome 9 (Xiong *et al*., 2021). The proximity between these genes suggests that their expression could be controlled by similar mechanisms. This study found two *CYP450* genes linked to PTX biosynthesis and identified 30 additional genes associated with known PTX biosynthesis genes in the established gene-to-gene co-regulatory network. Cheng *et al*. (2021) and Song *et al.* (2021) also published high-quality reference genomes for *Taxus wallichiana* and *Taxus yunnanensis*. These three chromosomal-scale reference genomes provide a valuable resource for investigating PTX biosynthesis, from the genes involved to the underlying mechanisms controlling their expression. This advance also unlocked new avenues for old datasets, by enabling metabolomic and proteomic approaches, together with the annotation of genes of interest in other *Taxus* species (Zhan *et al*., 2023).

## PTX biosynthesis breakthroughs

Following publication of the *Taxus* genomes (Cheng *et al*., 2021; Song *et al*., 2021; Xiong *et al*., 2021), research into the missing steps of PTX biosynthesis accelerated, leading to elucidation of the pathway genes (Table 1). The first breakthrough was a minimal gene set required for PTX biosynthesis, which identified five missing enzymes from the pathway, namely 2-oxoglutarate-dependent dioxygenase (epoxidase), taxane-9α-hydroxylase (T9αOH), taxane 1β-hydroxylase (T1βOH), taxane 9α-dioxygenase (T9α oxidase), and *Penicillium chrysogenum* phenylalanine-CoA ligase (PCL) (Y. Zhang *et al*., 2023). By utilizing published RNA sequencing (RNA-seq) datasets from Ramirez-Estrada *et al*. (2016), Liao *et al*. (2017), Kuang *et al*. (2019), Zhou *et al*. (2019), and Xiong *et al*. (2021), candidate genes were identified. This consisted of using the 13 previously characterized genes as bait, as genes involved in specialized biosynthetic pathways are often co-expressed (Mutwil, 2020). The top correlated genes across the datasets were determined and their putative annotations were assessed if they matched the criteria required for the missing steps of the PTX pathway. Screening of candidates was carried out by expressing them in *N. benthamiana* followed by metabolic analysis—production of taxadiene in *N. benthamiana* had been previously optimized (Li *et al.*, 2019). This approach has emerged as a key technology to uncover the nature of a given enzymatic pathway (Reed *et al*., 2023; Martin *et al*., 2024).

**Table 1.** List of all enzymes used for the PTX biosynthesis (Y. Zhang *et al.*, 2023) and for baccatin III biosynthesis (Jiang *et al*., 2024)

Abbreviation	Name	Reference
TXS	Taxadiene synthase	Wildung and Croteau (1996)
TAT	Taxadiene-5α-ol-*O*-acetyl transferase	Walker *et al.* (2000)
TBT	Taxane-2α-*O*-benzoyltransferase	Walker and Croteau (2000a)
DBAT	10-Deacetyl baccatin III-10-β-*O*-acetyltransferase	Walker and Croteau (2000b)
T13αOH	Taxane 13α-hydroxylase	Jennewein *et al*. (2001)
T10βOH	Taxane 10β-hydroxylase	Schoendorf *et al*. (2001)
BAPT	Baccatin III-3-amino-3-phenylpropanoyltransferase	Walker *et al.* (2002a)
DBTNBT	3ʹ-*N*-debenzoyl-2ʹ-deoxytaxol-*N*-benzoyltransferase	Walker *et al.* (2002b)
T2αOH	Taxane 2α-hydroxylase	Chau and Croteau (2004)
T7βOH	Taxane 7β-hydroxylase	Chau *et al.* (2004)
T5αOH	Taxadiene-5α-hydroxylase	Jennewein *et al*. (2004)
PAM	Phenylalanine aminomutase	Walker *et al*. (2004)
PCL	β-Phenylalanine coenzyme A ligase	Ramírez‐Estrada *et al.* (2016)
T2’αOH	Taxane 2ʹα-hydroxylase	Sanchez-Muñoz *et al.* (2020)
Epoxidase	2-Oxoglutarate-dependent dioxygenase	Y. Zhang *et al.* (2023)
T9αOH	Taxane 9α-hydroxylase	Y. Zhang *et al.* (2023)
T9α oxidase	Taxane 9α-dioxygenase	Y. Zhang *et al.* (2023)
T1βOH	Taxane 1β-hydroxylase	Y. Zhang *et al.* (2023)
PCL	*Penicillium chrysogenum* phenylalanine-CoA ligase	Y. Zhang *et al.* (2023)
T9αH	Taxane 9α-hydroxylase 1	Jiang *et al*. (2024)
TOT1	Taxane oxetanase 1	Jiang *et al*. (2024)

**Fig. 1.**
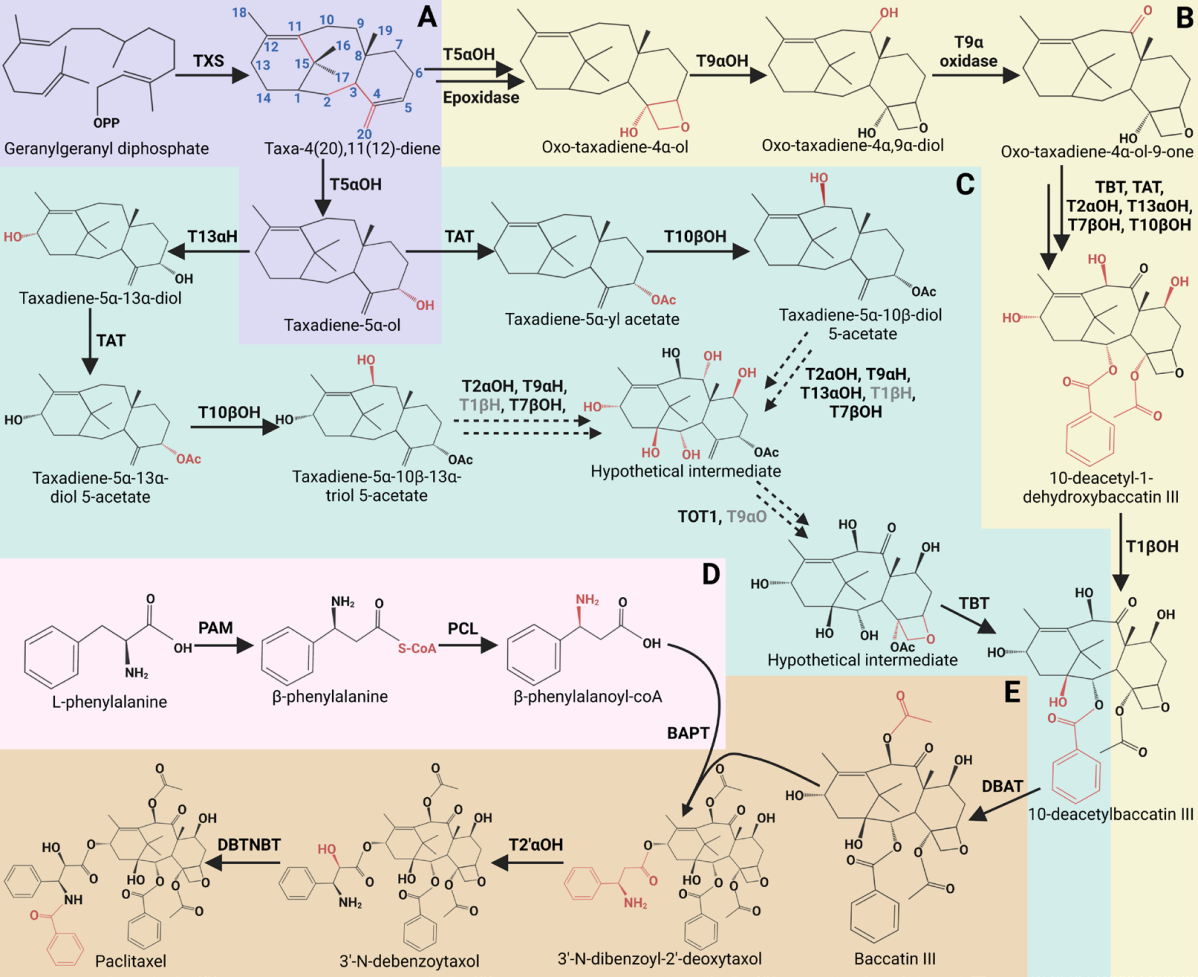
The current predicted pathway of PTX biosynthesis. (A) The first section of the pathway goes from geranylgeranyl diphosphate to taxadiene-5α-ol and is catalysed by TXS and T5αOH. Next are the steps from taxadiene-5α-ol to 10-deacetylbaccatin III via three possible routes. (B) The path shown by Y. Zhang *et al*. (2023) in their first module. (C) The two paths proposed by Jiang *et al.* (2024). The grey coloured enzymes represent proposed enzymes, as it is unsure which enzymes used by Jiang *et al.* (2024) fulfil the role of taxane 1β hydroxylase (T1βH) and taxane 9α oxidase (T9αO) in their proposed pathway. TOT1 probably carries out the function of a 4,20-epoxidase and a 4,5-oxomutase. (D) The side chain formation from l-phenylalanine to β-phenylalanoyl-CoA. (E) The end steps of PTX biosynthesis and attachment of the side chain going from 10-deacetylbaccatin III shown by Y. Zhang *et al*. (2023) in their second module. Jiang *et al*. (2024) observed that DBAT could be replaced by TAT for baccatin III production. Created with BioRender.com.

To support this approach, the PTX pathway was split into two modules due to its complexity and diminishing yield for each subsequent step (Y. Zhang *et al*., 2023). Four newly identified enzymes (epoxidase, T9αOH, T1βOH, and T9α oxidase) were expressed in *N. benthamiana*, together with the characterized enzymes for the first module, and the module ends with baccatin III production (Fig. 1B). The second module produces PTX after feeding with benzoic acid, l-phenylalanine, and baccatin III, by expressing the characterized enzymes—phenylalanine aminomutase (PAM), baccatin III-3-amino-3-phenylpropanoyltransferase (BAPT), 3ʹ-*N*-debenzoyl-2ʹ-deoxytaxol-*N*-benzoyltransferase (DBTNBT), and the more recently identified taxane 2ʹα-hydroxylase (T2ʹαOH); with two identified PCL genes, *TAAE16* from *Taxus* and *Pc21g30650* from *P. chrysogenum* (Koetsier *et al*., 2011)—in *N. benthamiana* (Fig. 1E). Y. Zhang *et al*. (2023) were able to produce 64.29 ng g^–1^ FW PTX using *Pc21g30650* and 29.39 ng g^–1^ FW using *TAAE16*. However, much progress is required to meet yields achieved with PCCs or natural harvest, 941.40 ng g^–1^ FW PTX from *Taxus* leaves (Y. Zhang *et al*., 2023). These results suggest that the minimal pathway is functional and that PTX could be produced in a heterologous host.

The second breakthrough identified two missing enzymes that enabled production of the intermediate baccatin III in *N. benthamiana* (Jiang *et al*., 2024) (Fig. 1C). This study utilized previous datasets (Xiong *et al*., 2021) to select candidate enzymes, which enabled the elucidation of taxane oxetanase 1 (TOT1) and taxane 9α-hydroxylase 1 (T9αH). These enzymes facilitated the assembly of baccatin III biosynthesis in *N. benthamiana* (Jiang *et al*., 2024). Taxane 10β-hydroxylase (T10βH) was found not to be essential for the oxidation at the C10 position, and 10-deacetyl baccatin III-10-β-*O*-acetyltransferase (DBAT) is not vital for C10 acylation of baccatin III, where taxadiene-5α-ol-*O*-acetyl transferase (TAT) was observed to fulfil this role. Also, taxane 13α-hydroxylase (T13αH) was shown to catalyse C13 hydroxylation with multiple substrates and oxidize taxadiene to different products, one being taxadiene-5α,10β,13α-triol. This promiscuity is seen in other enzymes of the pathway, leading to the complexity of PTX biosynthesis and its regulation (Mutanda *et al*., 2021), which may explain why the baccatin III yield achieved was low (~50 ng g^–1^). These studies have identified key resources and routes for paclitaxel and baccatin III production. However, further work is required for determining the optimal route to enable higher yields. With possible pathways identified, future studies can now focus on PTX biosynthesis regulation.

Lack of a functional gene set was a major limitation facing PTX production in heterologous hosts (Perez-Matas *et al*., 2023a). The newly elucidated routes provide new potential for the pathway to be assembled in microbial chassis, providing a cheap and efficient alternative for production. This is due to microbes avoiding unnecessary cellular metabolites, being easier to manipulate genetically, faster growing, and more stable than PCCs in bioreactors (Mutanda *et al*., 2021). However, PTX biosynthesis in microbial hosts needs to overcome the cytochrome P450 enzymes in the pathway (Pyne *et al*., 2019). For example, taxadiene-5α-hydroxylase (T5αOH) lacks specificity for reagents or products, causing poor expression and catalytic activity in heterologous hosts (Edgar *et al*., 2016; Sagwan‐Barkdoll and Anterola, 2017); it is also difficult to express the functional protein in *Escherichia coli* (Rouck *et al*., 2017). Model plants are being investigated as alternative heterologous hosts for PTX production, but these suffer from low yields and adverse effects on the plant growth (Besumbes *et al*., 2004; Li *et al.*, 2019). Consequently, *Taxus* PCCs remain the most attractive means of production, due to the ease of applying elicitors, such as MeJA, to increase PTX production (Yukimune *et al*., 1996), and the current work targeting rate-limiting steps (Perez-Matas *et al.*, 2023b) with new genes offering potential for manipulation.

## Regulation of PTX biosynthesis

Many avenues have been explored to improve PTX yields from PCCs from identification of high-yielding *Taxus* species (Zhou *et al*., 2019) and tissues (Lee *et al*., 2010) for generating PCCs, to optimization of media (Cusidó *et al.*, 2002). One of the main approaches for increasing PTX production is through manipulation of its regulation. With the publication of three *Taxus* genomes (Cheng *et al*., 2021; Song *et al*., 2021; Xiong *et al*., 2021) and functional pathways for PTX biosynthesis (Y. Zhang *et al*., 2023; Jiang *et al*., 2024), there is great potential for identification of new regulators. In this context, Zhan *et al.* (2023) created the first *Taxus* leaf metabolic single-cell atlas via MALDI-2 imaging (matrix-assisted laser desorption ionization mass spectrometry imaging) and single-cell transcriptional profiling of *Taxus marei* leaves. Here they identified four TFs (WRKY12, WRKY31, GT_2, and ERF13) that up-regulated expression of *DBAT* and two TFs (MYB17 and bHLH46) that down-regulated expression of *taxadienesynthase* (*TXS*) and *geranylgeranyl diphosphate synthase* (*GGPPS*). Regulation of PTX biosynthesis has mainly focused on the identification of elicitors and TFs, with nine elicitors and 22 TFs being found to activate different areas of the pathway, as well as 10 repressive TFs (Table 2). Regulation of PTX biosynthesis is probably considerably more complex given that a study by Tang *et al.* (2021) identified 2039 TFs and found that 974 of the TFs bound promoters in *Arabidopsis thaliana* for aliphatic glucosinolate biosynthesis (a PSM), 933 TFs of which also bound promoters from a central carbon pathway. This shows the complexity and scale of plant specialized metabolism and its associated regulators (Kliebenstein, 2023).

**Table 2.** All currently known elicitors and TFs which have been found to regulate PTX biosynthesis and their function

	Name	Function	Reference
**Elicitors**	Ag^+^	Activator	Zhang *et al.* (2000)
	Arachidonic acid	Activator	Srinivasan *et al.* (1996)
	Coranatine	Activator	Onrubia *et al.* (2013)
	Chitosan	Activator	Zhang *et al.* (2000)
	Cyclodextrins	Activator	Sabater‐Jara *et al.* (2014)
	Fungal elicitors	Activator	Ciddi *et al*. (1995)
	Methyl jasmonate	Activator	Yukimune *et al.*(1996)
	Phytosulfokine-α	Activator	Kim *et al*. (2006)
	Salicylic acid	Activator	Wang *et al*. (2004)
**Transcription factors**	TmbHLH13	Activator	Yu *et al.* (2022)
	TmbHLH14	Repressor	Zhan *et al*. (2023)
	TcERF12	Activator	Zhang *et al.* (2015)
	TmERF13	Activator	Zhan *et al*. (2023)
	TcERF15	Repressor	Zhang *et al.* (2015)
	*TmGT_2*	Activator	Zhan *et al*. (2023)
	*TmJAM1*	Repressor	Cui *et al.* (2019)
	*TmJAM2*	Repressor	Cui *et al.* (2019)
	TcJAMYC1	Repressor	Lenka *et al*. (2015)
	TcJAMYC2	Repressor	Lenka *et al*. (2015)
	TcJAMYC4	Repressor	Lenka *et al*. (2015)
	TmMYB3	Activator	Yu *et al.* (2020)
	TmMYB17	Repressor	Zhan *et al.* (2023)
	TcMYB29a	Activator	Cao *et al.* (2022)
	*TmMYB39*	Activator	Yu *et al*. (2022)
	*TmMYC2*	Activator	Cui *et al.* (2019)
	*TmMYC3*	Activator	Cui *et al.* (2019)
	*TmMYC4*	Activator	Cui *et al.* (2019)
	*TcMYC2a*	Activator	Zhang *et al.* (2018b)
	*TcWRKY1*	Activator	S. Li *et al.* (2013)
	*TcWRKY8*	Activator	Zhang *et al.* (2018a)
	Tm*WRKY12*	Activator	Zhan *et al*. (2023)
	*TcWRKY20*	Activator	Zhang *et al.* (2018a)
	*TcWRKY26*	Activator	Zhang *et al.* (2018a)
	Tm*WRKY31*	Activator	Zhan *et al*. (2023)
	*TcWRKY33*	Activator	Chen *et al.* (2021)
	*TcWRKY41*	Activator	Zhang *et al.* (2018a)
	*TcWRKY44*	Repressor	Zhang *et al.* (2018a)
	*TcWRKY47*	Activator	Zhang *et al.* (2018a)
	*TcWRKY52*	Repressor	Zhang *et al.* (2018a)
	BIS2	Activator	Sanchez-Muñoz *et al.* (2019a)
	TSAR2	Activator	Sanchez-Muñoz *et al.* (2019a)

TF- and elicitor-mediated regulation is one way in which PSMs are regulated, with recent research identifying the critical role of epigenetics (Zhao *et al*., 2023). Regulation of PSM production in PCCs through epigenetic modification is of particular concern as long-term *in vitro* conditions for PCCs are linked to production loss of PSMs (Tabata and Hiraoka,1976; Dougall *et al*.,1980; Kim *et al.*, 2004; L.Q. Li *et al.*, 2013). Sanchez-Muñoz *et al.* (2018) identified methylated cytosines on the *BAPT* promoter in its Y-patch region for 10-year-old (low-yield) *Taxus×media* cell cultures that were absent in the new (high-yield) cultures, implying a possible correlation between the loss of PTX production in old PCCs and DNA methylation at the promoters of genes involved in PTX biosynthesis. In addition, promoters for *GGPPS*, *TXS*, and *DBTNBT* were assessed for their methylation profiles in a new cell line compared with a 14-year-old cell line (Escrich *et al*., 2022). For *TXS* and *DBTNBT* promoters, DNA methylation was observed for PCCs maintained in *in vitro* conditions, reducing expression of these genes. Interestingly the *DBTNBT* promoter is heavily methylated before the transcription start site for both old and new cultures. This was not seen for the other two promoters, implying that the latter stages of PTX biosynthesis could be regulated by differential methylation (Escrich *et al*., 2022). In contrast, the *GGPPS* promoter was found to be protected, with no DNA methylation being detected. *GGPPS* encodes the enzyme that makes the precursor for the PTX biosynthesis pathway, geranylgeranyl diphosphate (Hefner *et al*., 1998). The preservation of this common precursor for primary metabolites in plants demonstrates that plant primary metabolism is preserved in long-term PCC conditions, whereas specialized metabolism is gradually lost.

DNA methylation-induced loss of PSMs in old PCCs is a considerable challenge for maintaining high-yield PTX lines. 5-Azacytidine, a DNA-demethylating agent, has shown promise for reversing DNA methylation in PCCs to increase PSM production (Tyunin *et al*., 2012; Zeng *et al*., 2019). Equally, the addition of 5-aza-2-deoxycytidine to *T. media* cells was seen to restore PTX production, although its toxicity is found to reduce biomass accumulation in the PCCs (L.Q. Li *et al.*, 2013). Targeted DNA demethylation seems promising for regulating epigenetic changes in promoter regions, showing success in *A. thaliana*. Gallego-Bartolomé *et al.* (2018) utilized a modified SunTag system (Tanenbaum *et al*., 2014) in a clustered regularly interspaced palindromic repeats (CRISPR)/CRISPR-associated protein 9 (CRISPR/Cas9)-targeted demethylation approach, along with the catalytic domain of human demethylase TEN-ELEVEN TRANSLOCATION1 (TET1) to demethylate their target. This could potentially be used to remove the DNA methylation observed by Escrich *et al.* (2022) in the *DBTNBT* promoter, restoring PTX production in the old cell line. Other successful approaches for targeted methylation in *A. thaliana* have replaced TET1 with the DRM methyltransferase catalytic domain (DRMcd) from *Nicotiana tabacum* (Papikian *et al*., 2019). Another SunTag variation demonstrated promise for demethylating histone marks (Fal *et al*., 2024, Preprint). Although no studies on the epigenetic regulation of histone marks in PTX production in *Taxus* cell cultures have been published, there is potentially a target here for regulation given the variation in PTX yield dependent on the tissue used for generating PCCs (Lee *et al*., 2010).

A promising study combined epigenetic regulation and chemical inhibition of phenylpropanoid biosynthesis, a competing pathway of PTX, to improve PTX production in *T. chinensis* cell cultures (Newton *et al*., 2023). Repression of phenylpropranoid biosynthesis with chemical inhibitors (phenylalanine, piperonylic acid, and caffeic acid) yielded a 3.5-fold increase in PTX accumulation compared with the control (Newton *et al*., 2023). This was partnered with repression of phenylalanine ammonia-lyase (PAL), the first committed step of phenylpropanoid biosynthesis, using the dCas9–SunTag system from Papikian *et al*. (2019). The repression successfully achieved a 25-fold increase in PTX accumulation in the methylated cell line PALg1 compared with the control (Newton *et al*., 2023). This is the first use of CRISPR in *Taxus* PCCs (Perez-Matas *et al*., 2023a), opening up new possibilities for gene editing of *Taxus* cell cultures. Furthermore, this highlights the functionality of dCAS9–SunTag systems in PCCs and demonstrates their suitability for use in reversing DNA methylation of plant specialized metabolism. This study shows a glimpse of the potential which can be unlocked through the different regulatory methods at play in the biosynthesis of PSMs.

## Conclusions

PTX and its derivative molecules remain a cornerstone for the treatment of an increasing variety of cancers, with new applications continuing to emerge, further promoting demand. The application of transcriptomic approaches with the completed *Taxus* genomes (Cheng *et al*., 2021; Song *et al*., 2021; Xiong *et al*., 2021) has already driven significant advances in our understanding of PTX biosynthesis with the identification of functional gene sets (Y. Zhang *et al.*, 2023; Jiang *et al*., 2024). While the pace of our understanding of PTX biosynthesis is accelerating, there is still much more to discover. Production of this key PSM in heterologous systems using synthetic biology approaches has made considerable progress; however, the yield of PTX remains low (Y. Zhang *et al.*, 2023). An optimized PTX gene set and order remains to be established for heterologous production to unlock their potential as commercial production platforms. Furthermore, the detailed regulatory mechanisms underpinning the control of PTX production in *Taxus* are yet to be fully elucidated, particularly the role of epigenetic modifications. This is key for solving the challenge of loss of PSMs in long-term PCCs (Sanchez-Muñoz *et al.*, 2019b). Nevertheless, exciting times clearly lie ahead for our understanding of the detailed molecular features associated with this important plant-derived pharmaceutical.
